# Upsides and downsides of a telecounselling model of integrated asthma management between general practitioners and specialists

**DOI:** 10.1002/clt2.12088

**Published:** 2022-02-03

**Authors:** Fabiana Furci, Marco Caminati, Sara Genovese, Sebastiano Gangemi, Gianenrico Senna

**Affiliations:** ^1^ Immunology Unit University Hospital Verona Italy; ^2^ Department of Clinical and Experimental Medicine School and Operative Unit of Allergy and Clinical Immunology, Policlinico “G. Martino”, University of Messina Messina Italy; ^3^ Department of Medicine University of Verona and Verona University Hospital Verona Italy; ^4^ Institute for Biomedical Research and Innovation, National Research Council of Italy (IRIB‐CNR), Messina Unit Messina Italy; ^5^ Asthma Centre and Allergy Unit University of Verona and Verona University Hospital Verona Italy

**Keywords:** asthma, asthma management, asthma severity, general practitioners, telemedicine, asthma, asthma management, asthmaschweregrad, Hausärtze, telemedizin

## Abstract

**Background:**

Asthma control, one of the most important goals in the management of asthmatic patients, requires good adherence to guidelines and support at a territorial level, in particular on the part of general practitioners (GPs). A territorial hospital alliance can become a strength in asthma management, where control by GPs can also be carried out through a spirometric examination.

**Methods:**

The realisation of a telecounselling model management of asthma between GPs and specialists was the aim of this study, to understand how to obtain good asthma control. A specific digital platform, the PneumoApp platform, was used for the insertion of clinical data and flow volume (F‐V) curves, performed in asthmatic patients by GPs, and for the subsequent evaluation of these data by specialists.

**Results:**

GPs have shown to be able to perform a check‐up of respiratory function well using a portable spirometer, but the analysis of the collected data showed that GP assessment of the severity level of asthma is incorrect in patients with moderate‐severe asthma.

**Conclusions:**

The effectiveness of a telecounselling collaboration between hospital and territory in the management of asthma patients can be improved by greater diffusion of the use of Global Initiative for Asthma (GINA) guidelines at a local level.

## 1 BACKGROUND

Asthma, a major public health and socio‐economic problem worldwide, is characterised by persistent airway inflammation, bronchial hyper‐responsiveness and a variable degree of airflow obstruction, with consequent respiratory symptoms, variable in terms of frequency and severity over time.^1^ Severity of asthma is often related to external factors and comorbidities, which influence lung function parameters, clinical symptoms and airway inflammation.^2^ Therefore, the concept of severity of asthma is quite complex and includes the level of therapy required to control symptoms and exacerbations. But the concept of severity of asthma is often more complex than this definition, and perhaps other variables such as clinical, functional and often endotype variables are often responsible for the failure to control asthma. Control of asthma, a fundamental goal of asthma management, is central in all guidelines and is based on symptom control and on future risk of adverse outcomes.^1^ Lack of asthma control still represents a great challenge for the management of this disease despite the availability of effective treatments. The causes of the lack of asthma control are various, from poor adherence to prescribed therapy to poor awareness of the disease or even difficulty in accessing specialist centres.^3^ Spirometry is often not performed in asthmatic patients, although it is considered mandatory in the diagnosis and management of asthma according to international guidelines.^1^ ^,^ ^4^, ^5^, ^6^

For every patient, management of asthma, according to Global Initiative for Asthma (GINA) guidelines, is based on assessment of asthma control, management of inhaler technique and adherence, and comorbidities that have an impact on the disease. In the assessment of the disease and of future risk of exacerbations, lung function and in particular forced expiratory volume in one second (FEV1), play an important role.^1^

One of the proposed strategies to overcome the issue is the involvement of first line healthcare professionals (HCPs—including general practitioners [GPs] and community pharmacies) in asthma management.^1^, ^7^ At a GP local level, a check‐up of asthmatic patients, through a spirometric examination could be carried out, allowing a diagnosis and better control of asthma that is not only clinical but also functional. A further support to overcome the above‐mentioned difficulty is the use of telemedicine, which represents the new frontier of clinical, diagnostic and therapeutic control, in a context in which doctors and patients are in two different locations while allowing remote collaboration between doctors in patient management.^8^, ^9^ Telemedicine allows to make a diagnosis and carry out the treatment of patients in remote locations, thanks to various telecommunication options, such as Internet monitoring, online patient reminder, text messages and e‐mail reminders. New technology has been developed and used to improve asthma control, becoming a powerful agent in the challenges in chronic disease management such as education, communication and adherence.^10^, ^11^, ^12^, ^13^, ^14^, ^15^

The use of telemedicine and tele‐healthcare in the management of asthma, in particular during the COVID‐19 pandemic, may become an instrument to realise the monitoring of asthma patients and a multidisciplinary approach, creating a network of collaboration between hospital and community.^16^

As reported by Caminati et al.^17^ GPs have a crucial role in the first approach with asthmatic patients who need specific assessment by specialists and, therefore, the recognition and sharing of common tools for the management of these patients is fundamental to obtain good asthma control in a multidisciplinary network between hospital and community.

This study aims to evaluate the sustainability of a collaboration project between hospitals and GPs in the management of asthma, based on telecounselling, highlighting how often at the base of the lack of asthma control there is an incorrect assessment of the level of asthma severity, leading to inappropriate therapy, which is not the one necessary to intervene correctly on the inflammatory process affecting the airways typical of asthma.

## 2 MATERIALS AND METHODS

This study, carried out from 1 March to 31 May 2019, involved specialists of the Asthma Centre and Allergy Unit, Verona University Hospital, Verona, and 38 GPs. A one‐day training course focused on both basic spirometry techniques and the use of the provided software was attended by all the GPs before the study initiation. All the patients with exemption 007, in particular 302 asthmatic patients, who arrived in the period of enrolment in their GP's office and who were invited to participate, accepted. No asthma patient refused to participate in the study.

Each GP performed the flow‐volume (F‐V) curve with a portable spirometer (Spirolab®/Mir) at their clinic and asked each participant to fill out a short 5‐item questionnaire regarding current treatment, use of short‐acting beta‐antagonists (SABA) and oral corticosteroids (OCS), number of exacerbations (defined by the need of a short course of OCS and/or antibiotics) and/or hospitalisations in the last year, smoking habit and definition of the current level of asthma severity according to the GINA guidelines. An allergological investigation was not performed in the enrolled patients.

A specific digital platform, the PneumoApp platform, was used for the insertion of the collected data and subsequent evaluation by both GPs and specialists. The specialists, by connecting through credentials to the PneumoApp platform, evaluated the F‐V curve performed and the data collected for each asthmatic patient by the GPs. The lung function tests performed by GPs were classified by specialists as either technically correct, interpretable or not interpretable. In addition, the specialists analysed both the total sample of patients enrolled in the study and two subgroups of patients based on the use or not of OCS.

In light of the F‐V curves performed and the clinical data collected, the specialists gave their assessment on each patient on the level of asthma severity, comparing it with that given by the GPs. The collected data were subjected to statistical analysis. In particular, data were reported as number of patients (percentage) for category variables, and as medians (interquartile range) or mean ± standard deviation for continuous variables with a non‐normal or normal distribution, respectively. Category variables were compared using the X2 test or the Fisher exact test, while continuous variables were assessed with the independent *t*‐test or the non‐parametric Mann–Whitney tests, as appropriate. All analyses were performed using IBM SPSS, version 25.0 (IBM Corp.). A *p*‐value of <0.05 was considered statistically significant.

## 3 RESULTS

The total sample of patients composed of 302 asthmatic patients was slightly more represented by females, with a mean age of 56 years.

Lung function data were available for 213 of the patients. Specialists divided the total sample of the patients into two subgroups: the subgroup of patients who did not use OCS and the subgroup of patients who used OCS. For one patient, information on OCS use was missing.

Subsequently, data collected were analysed as reported in Table 1.

**TABLE 1:** Baseline characteristics of asthmatic patients according to use of OCS

Variables	Total sample	Patients not using OCS	Patients using OCS	*p‐*value
Number of patients (%)	302 (100)	248 (82.4)	53 (17.6)	‐
Age, years	56 [22]	56 [22]	53 [26]	0.343
Male/female, %	51/49	52/48	47/53	0.504
FEV1, % predicted	93 ± 21	93 ± 20	93 ± 25	0.986
Smoking habit (no, current, former), %	49/27/24	48/26/26	54/29/17	0.532
Use of SABA, pack‐year				
0	39.5	39.1	41.5	0.756
1–2	43.5	44.8	37.7	
3–5	14.3	13.7	17	
≥6	2.7	2.4	3.8	
Exacerbations/year				
0	35.5	39.1	18.9	0.019
1	37.9	35.5	49.1	
≥2	26.6	25.4	32.1	
Hospitalizations/year				
0	95	96.4	88.7	0.036
1	4.7	3.2	11.3	
≥2	0.3	0.4	0	
Concordance of severity between GPs and specialists, %				
Mild	94	94	100	<0.001
Moderate	47	52	31	
Severe	39	25	50	

Abbreviations: FEV1, forced expiratory volume in one second; GPs, general practitioners; OCS, oral corticosteroids; SABA, short‐acting beta‐antagonists.

**FIGURE 1:**
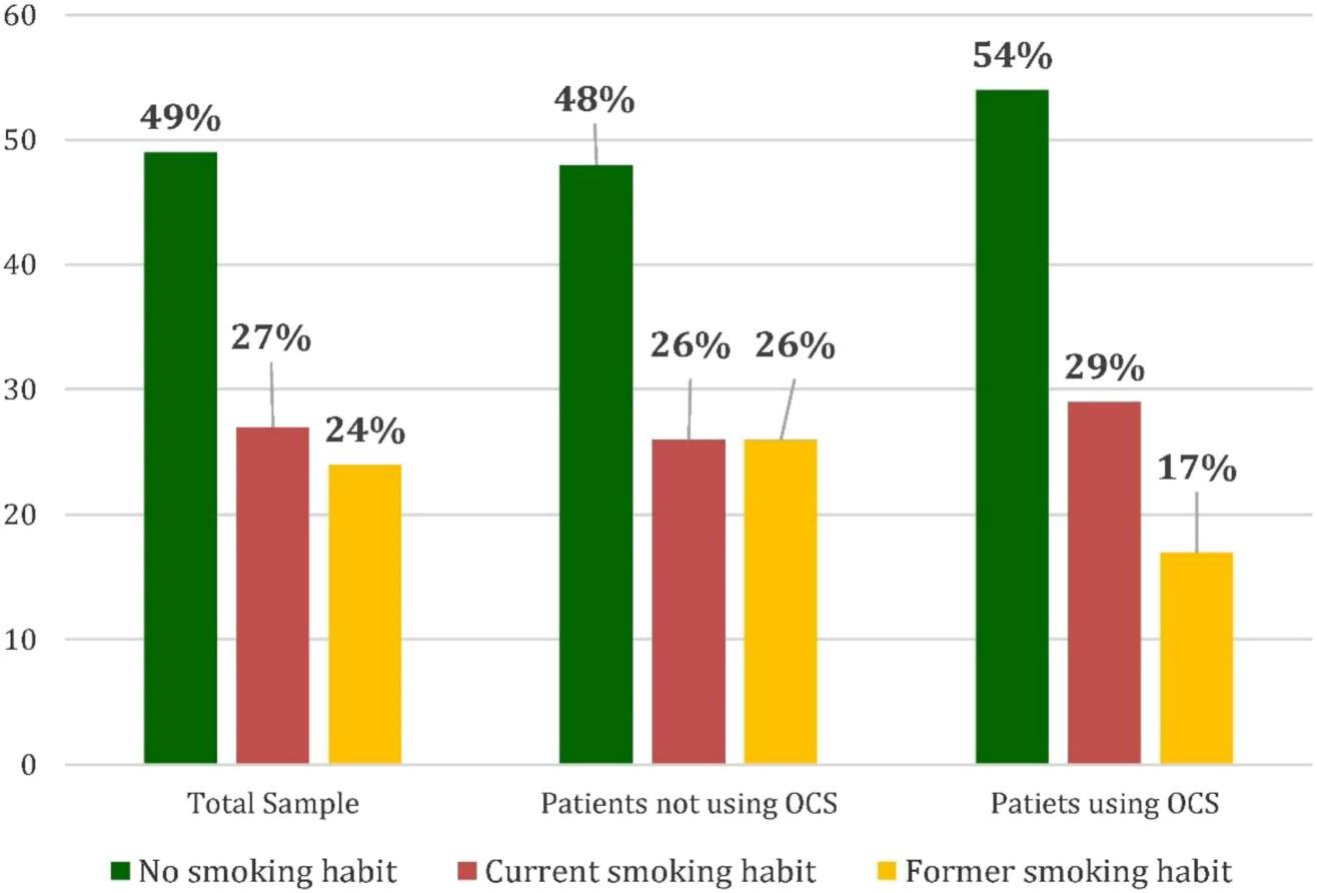
Smoking habit (no, current, former) %

Remembering the crucial role of smoking in asthmatic patients, analysing the total sample, as reported in Figure 1, 49% of patients had a no smoking habit, 27% were current smokers and 24% were former smokers. Analysing the groups of patients not using OCS and using OCS, we observed that:

- In patients not using OCS, 48% had a no smoking habit, 26% were current smokers and 26% were former smokers.
- In patients using OCS, 54% had a no smoking habit, 29% were current smokers and 17% were former smokers (Figure 1).

**FIGURE 2:**
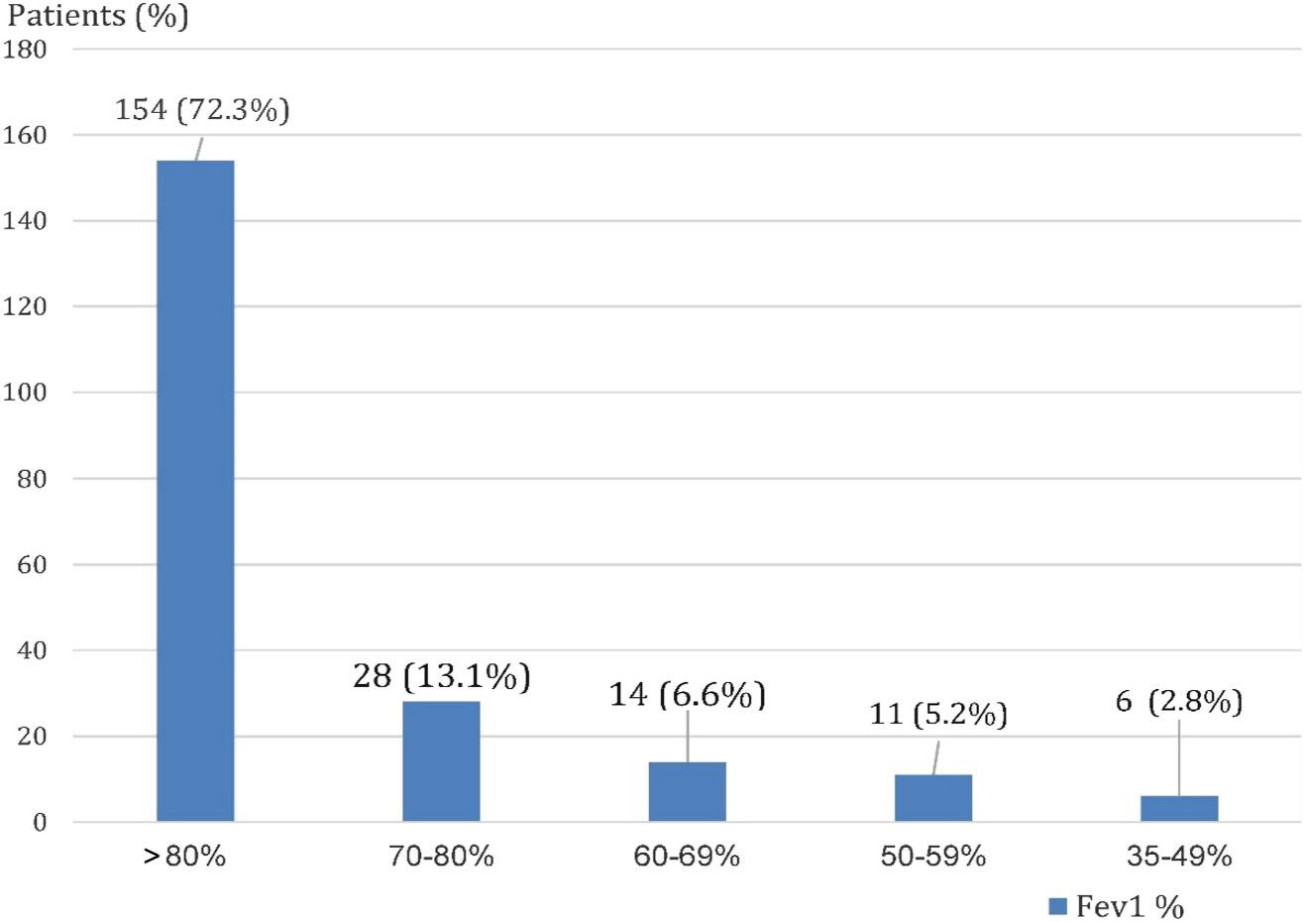
Values for forced expiratory volume in first second in the study

We also evaluated values for FEV1 in the 213 patients enrolled, with lung function assessed by GPs, finding that 72.3% of patients presented FEV1 > 80%, 13.1% presented FEV1 70%–80%, 6.6% of patients presented FEV1 60%–69%, 5.2% of patients presented FEV1 50%–59%, 2.8% of patients presented FEV1 35%–49% (Figure 2).

**FIGURE 3:**
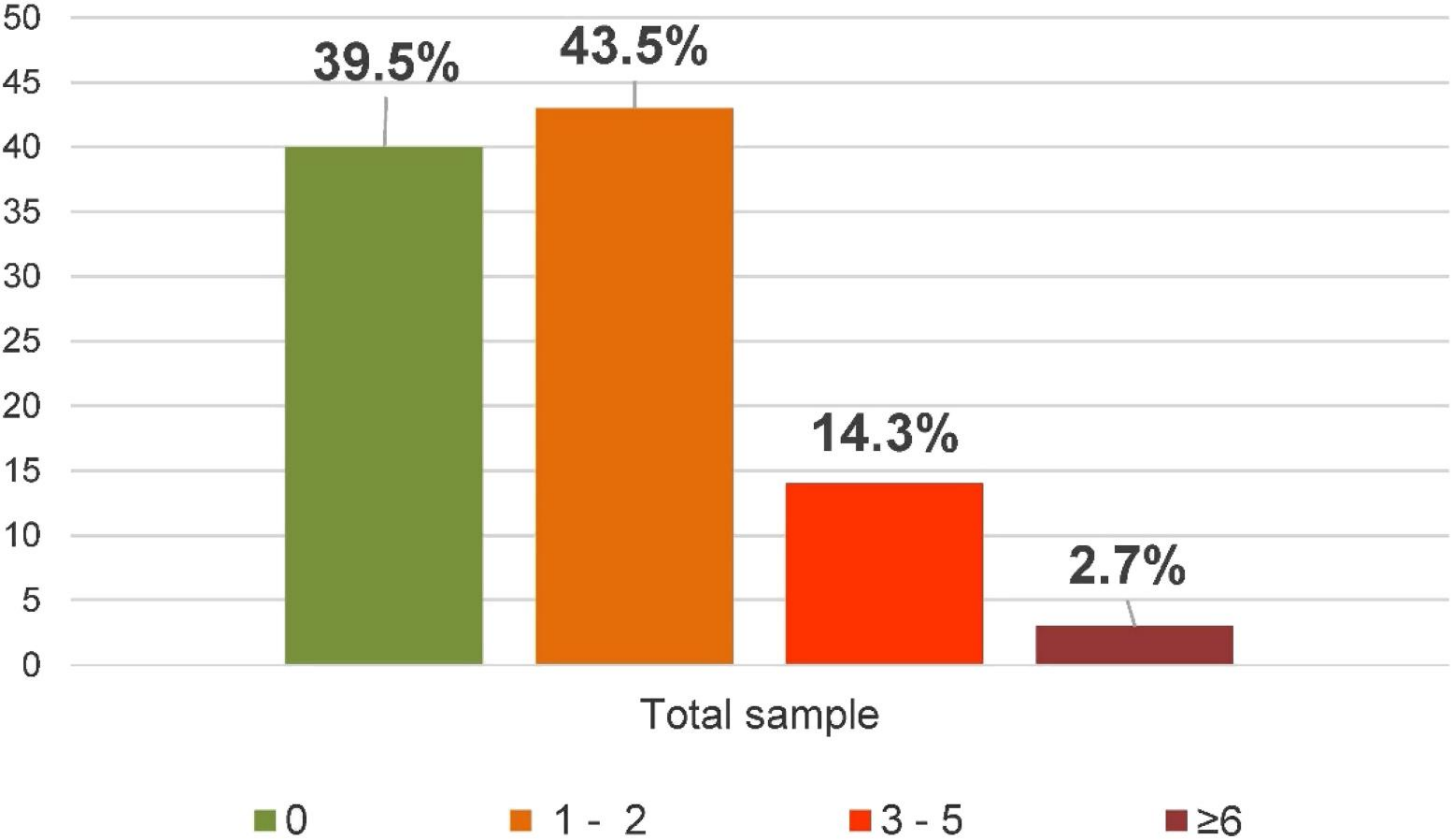
Use of short‐acting beta‐antagonists, pack‐year

Regarding the use of SABA, in particular, from analysis of the number of packs used in a year by patients enrolled in the study, it can be seen that 39.5% of patients did not use SABA, 43.5% used 1–2 pack‐year, 14.3% used 3–5 pack‐year, and 2.7% used ≥6 pack‐year (Figure 3).

**FIGURE 4:**
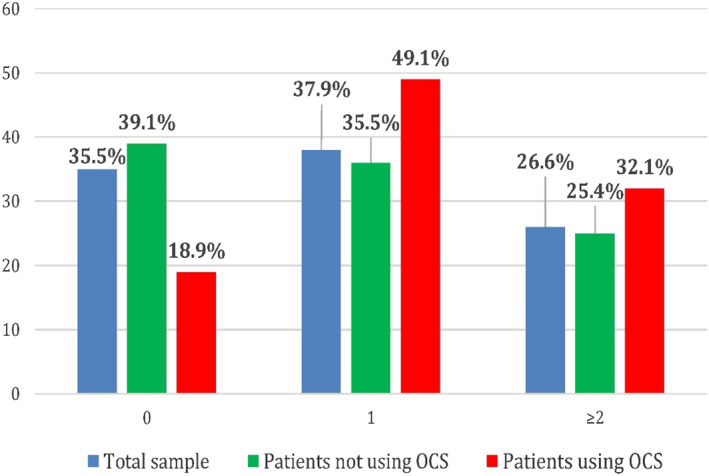
Exacerbations/year

**FIGURE 5:**
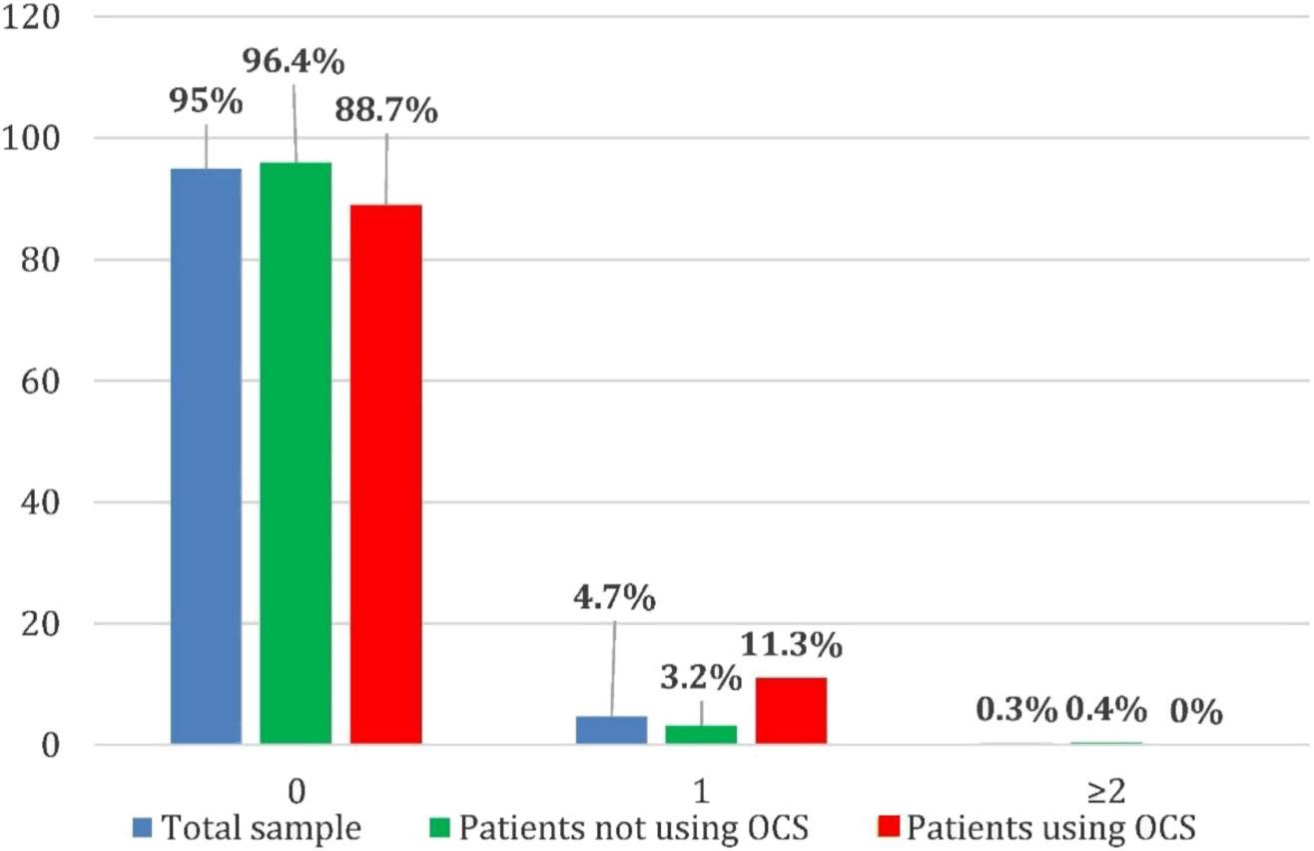
Hospitalisations/year

Analysing exacerbations/year and hospitalisations/year both on the total sample of patients and in the subgroups of patients that used OCS and did not use OCS, approximately 38% of patients had at least one exacerbation/year, whereas <5% referred at least one hospitalisation. No differences were observed between OCS users and OCS non‐users, except a significantly lower number of accesses to A&E or hospitalisations in the first group (Figures 4 and 5).

**FIGURE 6:**
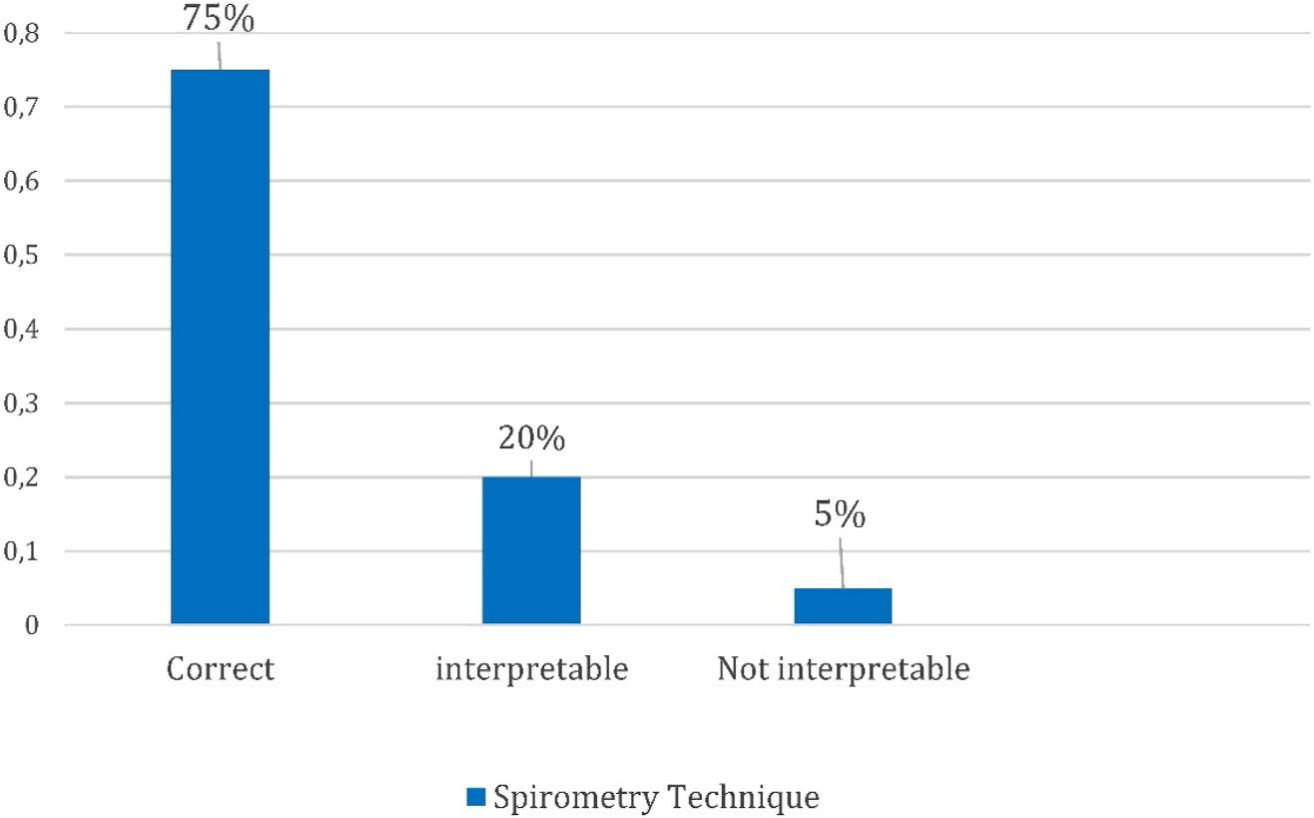
Specialists' classification of lung function performed by general practitioners

We analysed the F‐V curves performed by GPs with asthmatic patients who arrived at their clinics, observing that 75% of lung function tests were technically correct; 20% were technically interpretable; 5% were not technically interpretable (Figure 6).

**FIGURE 7:**
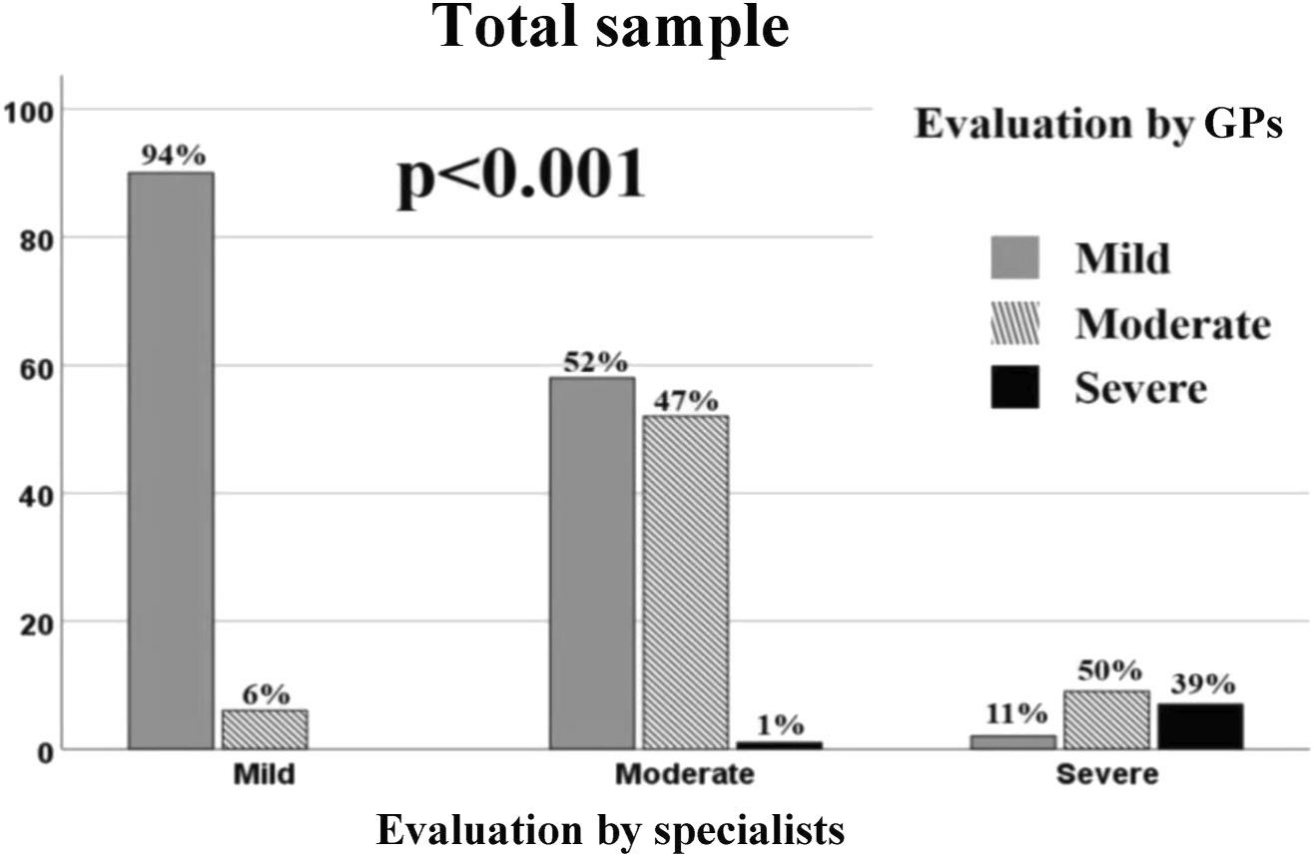
Evaluation of agreement between specialists and general practitioners (GPs) in the total sample on asthma severity in mild, moderate and severe asthma

An important part of our analysis included the comparison of asthma severity assigned by specialists and the severity level assessed by GPs in the enrolled patients, highlighting a lack of agreement in moderate asthma, and, in particular, severe asthma cases. Indeed, in moderate asthma, in which agreement between specialists and GPs was 47%, 52% was considered by GPs as mild asthma, instead 1% was considered by GPs as severe asthma. In severe asthma, in which agreement between GPs and specialists was 39%, GPs diagnosed 50% of patients as moderate asthma and 11% of patients as mild asthma (Figure 7).

**FIGURE 8:**
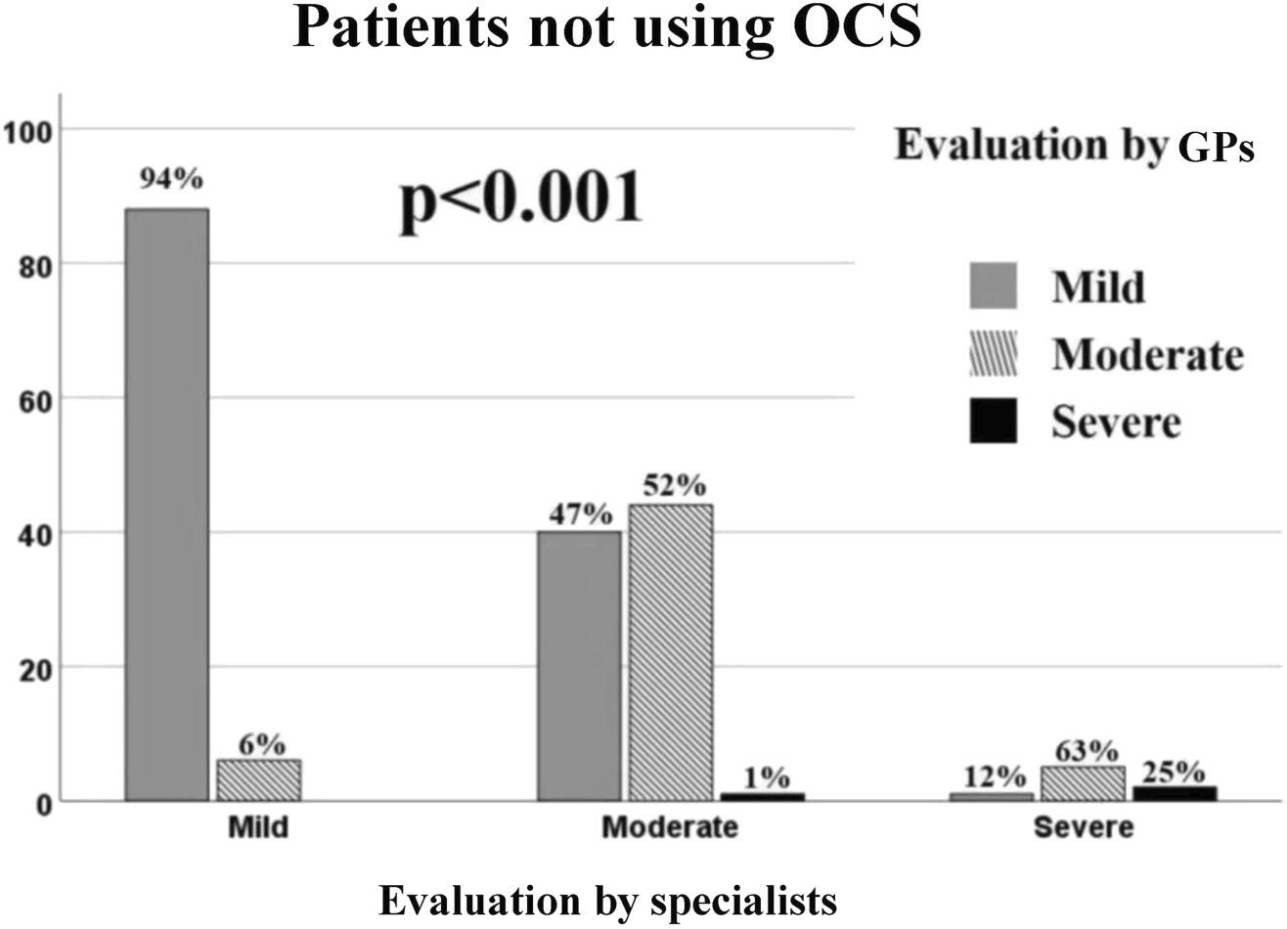
Evaluation of agreement between specialists and general practitioners (GPs) in patients not using oral corticosteroids (OCS) on asthma severity in mild, moderate and severe asthma

**FIGURE 9:**
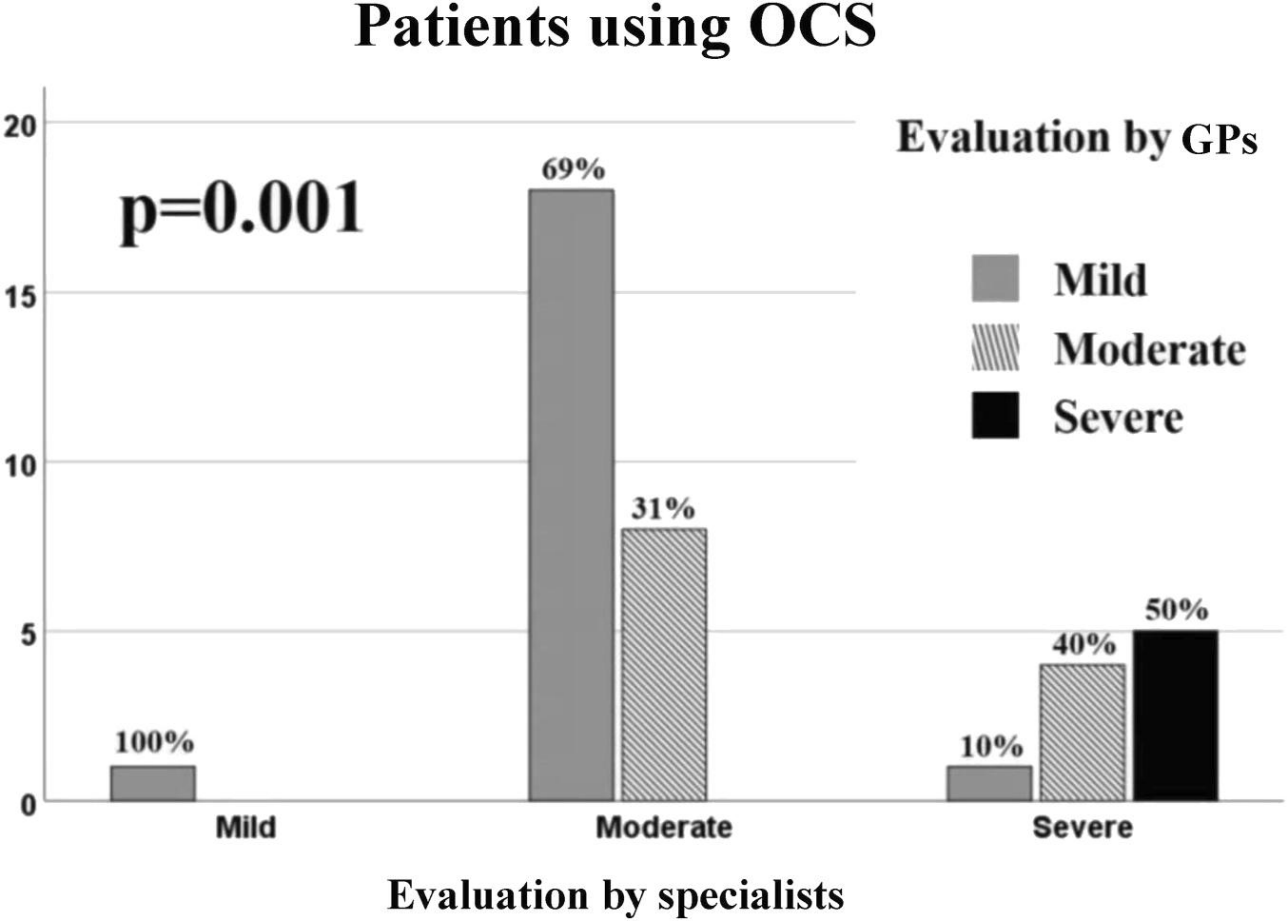
Evaluation of agreement between specialists and general practitioners (GPs) in patients using oral corticosteroids (OCS) on asthma severity in mild, moderate and severe asthma

Agreement between specialists and GPs was analysed also in the two subgroups, reporting a higher level of agreement on patients that used OCS (Figures 8 and 9).

## 4 DISCUSSION

This study reported a good approach from GPs in performing F‐V curves but not always a good interpretation of asthma severity level, which is not connected with FEV1 value. Indeed, the finding of normal F‐V curves, which is not a sign of good asthma control, could lead to an incorrect assessment of level of asthma severity and consequently to a milder therapeutic approach. Agreement between specialists and GPs was lower, above all in the total sample, regarding patients with moderate‐severe asthma.^18^

The low use of SABA, pack‐year, found in the enrolled patients, may be due to good asthma control and therefore a lower need of SABA or by careful adherence to GINA guidelines according to which, in the case of need, asthmatic patients can use the association ICS/LABA (long‐acting β2‐agonist) inhaled therapy, rather than only SABA that in itself does not confer an anti‐inflammatory response.

In the light of the higher use of OCS by asthmatic smokers rather than non‐smokers, from the study, smoking can be confirmed as a predictive factor of asthma exacerbations.^19^, ^20^

Regarding exacerbations/year and hospitalisations/year a significantly lower number of accesses to A&E and/or hospitalisations in the OCS user group suggests a possible preventive role of OCS therapy on hospitalisations but not on exacerbations.

The discrepancy between asthma severity classification between GPs and specialists leads to a consequent imbalanced treatment and to the need for GINA guideline implementation and increased knowledge at a territorial level, as previously reported in other studies.^21^, ^22^

A potential bias of the study might be the absence of an asthma control test (ACT) score, which, as reported in a previous study, is usually not performed by GPs as it is considered time consuming.^17^ Indeed, as reported by Braido et al., in a survey conducted among GPs and specialists to evaluate a series of physician‐related factors that can be related to uncontrolled asthma, the Asthma Control Test was used by only 20.15% of GPs and by 42.92% of specialists.

The treatment was evaluated in order to define the GINA stage. We did not report the specific treatment for each patient, as this is not included in the main aims of the study. However, also the lack of this information may represent another study limitation.

Our study demonstrated that once GPs are properly trained, they can effectively contribute to asthma patients' regular assessment by sharing lung function and clinical data with specialists. On the other hand, our results highlight the need for an alignment in terms of asthma severity classification, which is significantly discrepant between GPs and specialists, especially when considering severe asthma. It is not negligible in the light of the implications on treatment choices and overall asthma management. A suboptimal knowledge of the international guidelines may account for this.

## 5 CONCLUSIONS

This study highlights how the realisation of integrated management of asthma patients between hospital and the community is essential for achieving good asthma control, with territorial support, useful both for identifying patients with asthma and for controlling those who already suffer from the disease by performing specific anti‐asthma therapy.

Implementation of educational and digital programmes would be useful in improving the management of asthma and patients' asthma control, in particular in primary care. Better coordination between general practitioners and specialists would be helpful to improve asthma management, and the use of digital programmes could improve this multidisciplinary approach, for which the dissemination of guidelines at a primary level, in association with educational patient programmes, for example using a written asthma action plan, may determine better asthma control.

The downside of this integrated approach, as reported above, is the underestimation of asthma severity which might lead to uncontrolled asthma and an overuse of OCS that are responsible for severe systemic side effects.^23^, ^24^ An awareness and overcoming of the downside inherent in telecounselling can contribute to improving global asthma control and management.^25^, ^26^, ^27^, ^28^ This integrated hospital‐community model management, with the integration of digital healthcare tools, may become relevant in contexts where the emergence of a pandemic, such as that caused by SARS‐CoV‐2, will no longer have to be managed in crisis‐mode, but for longer periods, in particular for chronic diseases that require long‐term management and control.

## CONFLICT OF INTEREST

None of the authors has any conflicts of interest to declare.

## AUTHOR CONTRIBUTIONS

**Fabiana Furci:** Conceptualization; Data curation; Formal analysis; Methodology; Supervision; Validation; Visualization; Writing – original draft; Writing – review & editing. **Marco Caminati:** Conceptualization; Data curation; Formal analysis; Methodology; Supervision; Validation; Visualization; Writing – original draft; Writing – review & editing. **Sara Genovese:** Conceptualization; Data curation; Formal analysis; Methodology; Resources; Supervision; Validation; Visualization; Writing – review & editing. **Sebastiano Gangemi:** Conceptualization; Data curation; Formal analysis; Methodology; Project administration; Supervision; Validation; Visualization; Writing – review & editing. **Gianenrico Senna:** Conceptualization; Data curation; Formal analysis; Methodology; Project administration; Supervision; Validation; Visualization; Writing – original draft; Writing – review & editing.

## References

[1] Global Initiative for Asthma . Global Strategy for Asthma Management and Prevention; 2020. http://www.ginasthma.org

[2] Taylor DR , Bateman ED , Boulet L‐P , et al. A new perspective on concepts of asthma severity and control. Eur Respir J. 2008;32:545‐554. 18757695 10.1183/09031936.00155307

[3] Caminati M , Vaia R , Furci F , Guarnieri G , Senna G . Uncontrolled asthma: unmet needs in the management of patients. J Asthma Allergy. 2021;3(14):457‐466. 10.2147/JAA.S260604 PMC8104981 33976555

[4] Aaron SD , Vandemheen KL , FitzGerald JM , et al. Reevaluation of diagnosis in adults with physician‐diagnosed asthma. J Am Med Assoc. 2017;317:269‐279. 10.1001/jama.2016.19627 28114551

[5] Miller MR , Hankinson J , Brusasco V , et al. Standardisation of spirometry. Eur Respir J. 2005;26:319‐338. 16055882 10.1183/09031936.05.00034805

[6] Pellegrino R , Viegi G , Brusasco V , et al. Interpretative strategies for lung function tests. Eur Respir J. 2005;26:948‐968. 16264058 10.1183/09031936.05.00035205

[7] Caminati M , Cegolon L , Bacchini M , et al. The potential role of local pharmacies to assess asthma control: an Italian cross‐sectional study. BMC Publ Health. 2021;21:19. 10.1186/s12889-020-10080-1 PMC7784353 33402150

[8] de Zambotti M , Cellini N , Goldstone A , Colrain IM , Baker FC . Wearable sleep technology in clinical and research settings. Med Sci Sports Exerc. 2019;51:1538‐1557. 30789439 10.1249/MSS.0000000000001947 PMC6579636

[9] Greiwe J , Nyenhuis SM . Wearable technology and how this can be implemented into clinical practice. Curr Allergy Asthma Rep. 2020;20:36. 32506184 10.1007/s11882-020-00927-3 PMC7275133

[10] Morrison D , Wyke S , Saunderson K , et al. Findings from a pilot randomised trial of an asthma internet self‐management intervention (RAISIN). BMJ Open. 2016;6:e009254. 10.1136/bmjopen-2015-009254 PMC4874112 27173807

[11] Newhouse N , Martin A , Jawad S , et al. Randomised feasibility study of a novel experience‐based internet intervention to support self‐management in chronic asthma. BMJ Open. 2016;6:e013401. 10.1136/bmjopen-2016-013401 PMC5223671 28031210

[12] van der Meer V , van Stel HF , Detmar SB , Otten W , Sterk PJ , Sont JK . Internet‐based self‐management offers an opportunity to achieve better asthma control in adolescents. Chest. 2007;132:112‐119. 17400674 10.1378/chest.06-2787

[13] Rasmussen LM , Phanareth K , Nolte H , Backer V . Internet‐based monitoring of asthma: a long‐term, randomized clinical study of 300 asthmatic subjects. J Allergy Clin Immunol. 2005;115:1137‐1142. 15940125 10.1016/j.jaci.2005.03.030

[14] Rhee H , Allen J , Mammen J , Swift M . Mobile phone‐based asthma self‐management aid for adolescents (mASMAA): a feasibility study. Patient Prefer Adherence. 2014;214:63‐72. 10.2147/PPA.S53504 PMC3891581 24470755

[15] Perry TT , Rettiganti MR , Bian J , et al. Utilization and outcomes associated with mobile‐based asthma action plans compared to paper asthma action plans among adolescents. J Allergy Clin Immunol. 2016;137:AB100.

[16] Mustafa SS , Yang L , Mortezavi M , Vadamalai K , Ramsey A . Patient satisfaction with telemedicine encounters in an allergy and immunology practice during the coronavirus disease 2019 pandemic. Ann Allergy Asthma Immunol. 2020;125:478‐479. 32585178 10.1016/j.anai.2020.06.027 PMC7306705

[17] Caminati M , Magnoni MS , Rizzi A , et al. Asthma management among different specialists: results from a national Italian survey. Eur Ann Allergy Clin Immunol. 2014;46:74‐82. 24739126

[18] Derom E , van Weel C , Liistro G , et al. Primary care spirometry. Eur Respir J. 2008;31:197‐203. 18166597 10.1183/09031936.00066607

[19] Thomson NC , Chaudhuri R , Livingston E . Asthma and cigarette smoking. Eur Respir J. 2004;24:822‐833. 15516679 10.1183/09031936.04.00039004

[20] Polosa R , Thomson NC . Smoking and asthma: dangerous liaisons. Eur Respir J. 2013;41:716‐726. 22903959 10.1183/09031936.00073312

[21] Baldacci S , Simoni M , Maio S , et al. Prescriptive adherence to GINA guidelines and asthma control: an Italian cross‐sectional study in general practice. Respir Med. 2019;146:10‐17. 30665506 10.1016/j.rmed.2018.11.001

[22] Braido F , Baiardini I , Stagi E , Piroddi MG , Balestracci S , Canonica GW . Unsatisfactory asthma control: astonishing evidence from general practitioners and respiratory medicine specialists. J Investig Allergol Clin Immunol. 2010;20:9‐12. 20232768

[23] Bloechliger M , Reinau D , Spoendlin J , et al. Adverse events profile of oral corticosteroids among asthma patients in the UK: cohort study with a nested case‐control analysis. Respir Res. 2018;19:75. 29699563 10.1186/s12931-018-0742-y PMC5921395

[24] Volmer T , Effenberger T , Trautner C , Buhl R . Consequences of long‐term OCS therapy and its side effects in severe asthma in adults – a focused review of the impact data in the literature. Eur Respir J. 2018;52:1800703. 30190274 10.1183/13993003.00703-2018

[25] Wu AC , Rehman N , Portnoy J . The good, the bad, and the unknown of telemedicine in asthma and allergy practice. J Allergy Clin Immunol Pract. 2019;7:2580‐2582. 31706487 10.1016/j.jaip.2019.08.017

[26] Keswani A , Brooks JP , Khoury P . The future of telehealth in allergy and immunology training. J Allergy Clin Immunol Pract. 2020;8:2135‐2141. 32426217 10.1016/j.jaip.2020.05.009 PMC7233253

[27] Chongmelaxme B , Lee S , Dhippayom T , Saokaew S , Chaiyakunapruk N , Dilokthornsakul P . The effects of telemedicine on asthma control and patients' quality of life in adults: a systematic review and meta‐analysis. J Allergy Clin Immunol Pract. 2019;7:199‐216. 30055283 10.1016/j.jaip.2018.07.015

[28] Portnoy JM , Waller M , De Lurgio S , Dinakar C . Telemedicine is as effective as in‐person visits for patients with asthma. Ann Allergy Asthma Immunol. 2016;117:241‐245. 27613456 10.1016/j.anai.2016.07.012

